# Mild chronic hypoxia and the brain: an ambiguous relationship

**DOI:** 10.1186/s12974-025-03652-8

**Published:** 2026-01-23

**Authors:** Magdalena Wszędybył-Winklewska, Ewelina Czuba-Pakuła, Krzysztof S. Malinowski, Monika Waśkow, Katarzyna M. Michalak, Paweł J. Winklewski

**Affiliations:** 1 Department of Neurophysiology, Neuropsychology and Neuroinformatics, Medical University of Gdańsk, Gdańsk, Poland; 2 Division of Anatomy and Neurobiology, Medical University of Gdańsk, Gdańsk, Poland; 3 Institute of Health Sciences, Pomeranian University in Słupsk, Słupsk, Poland; 4 Department of Neurophysiology, Neuropsychology and Neuroinformatics, Medical University of Gdansk, Tuwima Str. 15, Gdansk, 80-210 Poland

**Keywords:** Mild hypoxia, Blood–brain barrier, Blood–spinal cord barrier, Mild brain inflammation, Mild systemic inflammation

## Abstract

Mild hypoxia is a common condition encountered in various situations, such as high-altitude living, respiratory diseases, anaemia, and certain cardiovascular disorders. At the same time, mild hypoxia often goes unnoticed and can lead to long-term brain impairments. Understanding the subtle effects of mild hypoxia on the brain is essential for early diagnosis and intervention to prevent serious neurological and psychiatric complications. The pathophysiology of mild chronic hypoxia is complex; nevertheless, based on the literature, two main pathways can be distinguished. The first involves the direct effects of mild hypoxia on the brain and spinal cord, while the second involves the influence of enhanced systemic inflammation. In this narrative review, we discuss the current understanding of the effects of mild hypoxia on brain metabolism, cellular models, perfusion, the blood–brain barrier (BBB), the blood–spinal cord barrier (BSCB) and the brain-cerebrospinal fluid barrier (BCSFB). We explore the intricate relationship between peripheral inflammation induced by hypoxia and the BBB/BSCB/BCSFB, shedding light on the underlying mechanisms and their potential implications for central nervous system health.

**Graphical abstract:**

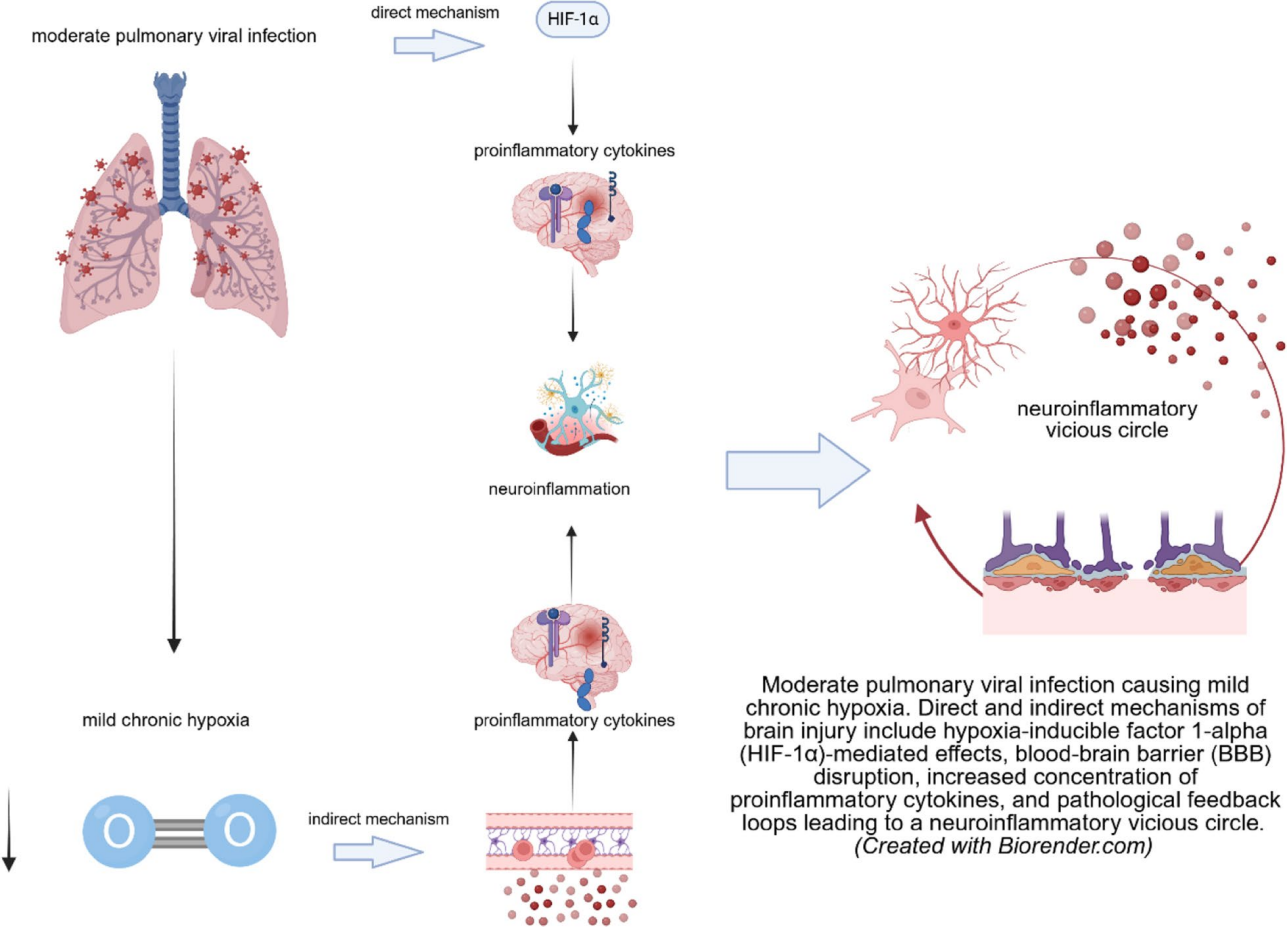

## Introduction

In recent years, there has been a notable increase in research examining the effects of mild chronic hypoxia on the brain. The relationship between the brain and mild chronic hypoxia is complex, multifaceted, and incompletely understood. Mild chronic hypoxia affects cerebral perfusion, metabolism, neural plasticity, mitochondrial function, and the integrity of the blood-brain barrier (BBB) and blood-spinal cord barrier (BSCB). Importantly, these effects can be either beneficial or detrimental depending on the severity, duration, and pattern of hypoxic exposure, as well as the baseline physiological state of the tissue [1, 2].

Mild hypoxia occurs in various common conditions, including high-altitude living, mild respiratory disorders such as chronic obstructive pulmonary disease, anaemia, and certain cardiovascular conditions [3–14]. While mild hypoxia can activate adaptive responses that may enhance cellular resilience [15, 16], it often goes undetected and can lead to long-term cognitive impairments and neurological disorders when chronic or severe [17]. It is often challenging to distinguish between hypoxia exposures that trigger beneficial adaptations and those that cause cumulative damage [18]. Understanding both the protective and harmful effects of mild hypoxia on the brain is crucial for early diagnosis, appropriate intervention, and the development of hypoxia-based therapeutic strategies while preventing serious neurological complications.

Mild chronic hypoxia is a potent regulator of neural stem cell (NSC) function and neural plasticity, with effects that can be both adaptive and maladaptive [19, 20]. Under controlled conditions, it promotes NSC self-renewal, enhances differentiation into neurons and glial cells, and activates adaptive molecular pathways, such as those mediated by hypoxia-inducible factor 1-alpha (HIF-1α) [21, 22]. These responses can contribute to improved cellular survival, metabolic reprogramming, and the maintenance of BBB integrity [23]. However, severe or prolonged hypoxia can also cause oxidative stress, mitochondrial dysfunction, and cellular damage [24, 25]. The term “mild chronic hypoxia” itself requires careful interpretation, as exposure conditions that allow survival through adaptive mechanisms in experimental models may still involve significant initial pathological responses [26]. The therapeutic potential of NSCs under hypoxic conditions and their relevance to neurodegenerative diseases, mitochondrial dysfunction, and central nervous system injuries must therefore be balanced against potential risks [27, 28]. 

The therapeutic potential of controlled mild chronic hypoxia has emerged as an intriguing area of research, though it requires careful consideration of risks and benefits [29, 30]. For instance, hypoxic preconditioning has been shown to suppress the development of experimental autoimmune encephalomyelitis, a murine model of multiple sclerosis [31–33], and, in a pre-existing relapsing-remitting model of experimental autoimmune encephalomyelitis, it accelerated clinical recovery [34]. Chronic mild hypoxia can augment vascular density and delay both the onset and severity of disease [35]. However, these promising preclinical findings must be interpreted with caution. The hypoxia exposures used in rodent studies (typically 8–10% oxygen (O_2_)) correspond to extreme altitude conditions for humans (~ 5500–6000 m) [36, 37], and initial exposure causes marked pathological events, including substantial cell death, before adaptive mechanisms emerge [26]. 

In this narrative review, we discuss the current understanding of mild hypoxia effects on cerebral perfusion and metabolism, cellular models, vascular remodelling, and the BBB and BSCB. The aim of this review is to synthesise current data on the neurovascular unit and the cellular effects of mild chronic hypoxia, critically examining both protective and detrimental outcomes. We identify key mechanisms that may have significant clinical relevance while acknowledging the complexity and context-dependency of hypoxic responses. We explore the delicate balance between protective and detrimental effects of mild hypoxia-induced inflammation in the brain, highlighting the underlying mechanisms, temporal dynamics, and their potential implications for central nervous system health.

We address the critical need for a precise definition of “mild chronic hypoxia” across different experimental systems and discuss the challenges in translating findings from animal models to clinical applications.

## Definition of chronic mild hypoxia across experimental systems

Defining “chronic mild hypoxia” requires several clarifications: oxygen concentration and exposure duration, differentiation between in vivo and in vitro models, and precise characterization of oxygen partial pressure (pO_2_) changes across physiological compartments. This section aims to discuss definitions as well as the tension between nomenclature (“mild”) and measured oxygen tissue concentrations.

To understand what “mild hypoxia” actually means, the following normal reference values are essential (Table 1). For instance, at 8% O₂ normobaric conditions, inspired oxygen pressure (PiO₂) is approximately 60 mmHg and alveolar pO₂ approximately 53 mmHg—values pathologically low and comparable to levels in severe acute respiratory distress syndrome (ARDS) patients [1, 2].

**Table 1:** Reference values for hypoxia across various compartments

Compartment	Normal (21% O₂)	Mild hypoxia (8–10% O₂)	Clinical reference
Inspired (PiO₂)	160 mmHg	~ 60 mmHg	—
Alveolar (PAO₂)	~ 100 mmHg	~ 53 mmHg	Severe ARDS threshold < 60 mmHg
Arterial (PaO₂)	95–100 mmHg	< 60 mmHg	Severe hypoxemia classification
Cerebral tissue (PtO₂)	~ 50 mmHg	~ 6.4 mmHg	Still above oxidative failure threshold (~ 1 mmHg)

inspired oxygen pressure (PiO₂); alveolar oxygen pressure (PAO2); arterial oxygen pressure (PaO₂); cerebral tissue oxygen pressure (PtO₂); ARDS - acute respiratory distress syndrome

In rodent studies, chronic mild hypoxia is defined as continuous normobaric exposure to 8–10% O₂ for ≥ 3 days [1, 15, 23, 27]. The minimum exposure duration of 3 days reflects the time required for adaptive signalling to emerge; shorter exposures (24–48 h) produce predominantly pathological responses. However, the hypoxia duration window differs across species and experimental models: studies in mice have reported adaptive responses as early as 24 h [16], while larger mammals may require 5–7 days for comparable adaptation.

Based on temporal response patterns, the following changes are observed:

- 24–48 h: marked pathological events occur, with 30–60% cell death in choroid plexus and ependymal layer [1], 10-fold elevation in plasma cytokines (interleukin – 6 (IL-6), interleukin − 13 (IL-13), vascular endothelial growth factor (VEGF), granulocyte-macrophage colony-stimulating factor (GM-CSF)) [27], BBB disruption with albumin leakage into cerebrospinal fluid (CSF) [1], retinal edema [16], and oxidative stress markers doubled (NADPH oxidase 4 (NOX4), circulating hydroperoxides) [18].
- 3–7 days: adaptive mechanisms predominate with HIF-1α stabilization and nuclear translocation [10], angiogenesis initiation [19], neurotrophic factor upregulation (VEGF, brain – derived neurotrophic factor (BDNF), erythropoietin (EPO) [28], and enhanced neural stem cell proliferation and differentiation [20, 27].
- 2–4 weeks: full adaptation is achieved with 50% increase in capillary density [19, 24], BBB integrity restoration [1], sustained NSC proliferation [20], enhanced neuroplasticity [25], and HIF-1α target gene expression stability [10].

Consequently, 8–10% O₂ qualifies as “mild chronic hypoxia” only when considering long-term adaptive changes and organism survival, not the acute pathological phase. Findings vary substantially by duration, and comparisons across studies must account for exposure timing [26]. 

Cultured NSC, endothelial cells, and neurons are exposed to 2.5–5.0% O₂ to maintain viability while stabilizing HIF-1α [29, 30]. This concentration range is selected based on evidence that severe hypoxia (< 1% O₂) induces quiescence and apoptosis, while normoxic conditions (20% O₂) induce differentiation and mitotic arrest [30]. 

Cell culture at 2.5–5.0% O₂ produces tissue PO₂ of approximately 5–15 mmHg. While the in vitro oxygen concentration (2.5–5.0%) is lower than the inspired oxygen in most normobaric animal models (8% O₂), the resulting tissue pO₂ in vivo (~ 6.4 mmHg at 8% O₂) falls within the range observed in vitro, though achieved under different diffusion conditions [1]. These differences in oxygen diffusion conditions arise because in vitro lack:

- systemic compensation. Cell cultures lack altered hemoglobin concentration, modified ventilation, enhanced cardiac output, and other systemic adaptations present in living organisms,
- barrier filtering, the BBB, which modulates immune cell and cytokine entry in vivo, is absent in culture,
- spatial heterogeneity, oxygen gradients vary between periarteriolar and perivenous spaces in vivo; in vitro systems apply uniform oxygen tension.

Consequently, cellular findings are useful to establish molecular mechanisms evoked by a hypoxic environment (such as HIF-1α pathway activation or metabolic reprogramming) but require validation in vivo before extrapolation to organism-level effects and disease states.

Prolonged exposure to altitudes ≥ 2500 m evokes hypobaric hypoxia with atmospheric pO₂ ~70 mmHg and arterial pO₂ ~60–70 mmHg after acclimatization [36]. This represents the closest natural human equivalent, although substantially different from normobaric animal models due to barometric pressure reduction and overall organism adaptation due to acclimatization [1]. At high altitude, the normal physiological response includes increased ventilation, elevated hemoglobin concentration and hematocrit, enhanced cerebral blood flow (CBF), and EPO production; such mechanisms are absent or minimized in sealed normobaric hypoxia chambers. [18] Physiological adaptation requires 2–3 weeks and progresses through increased minute ventilation, reduced partial pressure of carbon dioxide (PaCO₂) (respiratory alkalosis), and compensatory metabolic adjustments [37, 38]. 

Recent therapeutic protocols employ 10–16% O₂ (not 8%) in intermittent cycles (2–5 min hypoxia/normoxia cycles, 3–7 sessions weekly, 30–240 min duration, 2–6 weeks total) [26]. Systematic reviews indicate that this lower concentration range is deliberately selected to activate adaptive signaling (HIF-1α, VEGF, BDNF, EPO upregulation) while minimizing acute tissue damage and inflammatory burden observed at 8% O₂ [39]. The selection of 10–16% over 8% O_2_ reflects empirical data review: 75% of human studies with moderate hypoxia (10–16% O₂) show cognitive and neurological benefits, whereas protocols using severe hypoxia (< 8% O₂) show higher rates of adverse effects [26]. 

Chronic mild hypoxia in rodents represents a physiological paradox: exposure to 8–10% O₂ produces arterial oxygen tensions (PaO₂ <60 mmHg) that would be classified as severe hypoxemia by human clinical standards [1], yet organisms survive through compensatory mechanisms. The designation “mild chronic hypoxia” reflects the sublethal, chronically tolerable nature of exposure rather than arterial severity. This terminology originated in early hypoxia research, where “mild,” “moderate,” and “severe” were operationally defined by survival and adaptation rather than blood gas criteria [17]. 

The pathological response in initial acute phases (24–48 h) transitions to predominantly adaptive responses by 3 + weeks [1, 16, 18]. Neither characterization alone captures the full picture; outcome depends critically on observation timing, tissue type, and individual susceptibility.

## Cerebral perfusion, blood oxygenation, and oxidative metabolism

The central nervous system, because of its complex structure and continuous demand for O_2_, is particularly sensitive to interruptions in O_2_ supply. Neural signalling, including the generation of action potentials and the constant re-establishment of ion gradients, requires a continuous supply of energy. While ion pumping is the largest energy consumer in the brain, other processes such as calcium transport, neurotransmitter recycling, and vesicle repacking also rely on adenosine triphosphate (ATP) [40, 41]. 

ATP is synthesised from adenosine diphosphate (ADP) by coupling the energy-demanding conversion of ADP to ATP with the energy-releasing breakdown of glucose and O_2_ to carbon dioxide (CO_2_) and water [42–44]. Importantly, the tissue O_2_/CO_2_ ratio is a key factor in maintaining the ATP/ADP balance [43, 44]. This understanding of the brain’s energy metabolism has several implications for understanding the dynamics of CBF in mild hypoxia.

During incremental ascent to high altitude, CBF regulation is governed by a critical “threshold phenomenon" [45–48], where competing chemostimuli, such as hypoxia-induced vasodilation and hypocapnia-induced vasoconstriction, dynamically interact to modulate brain perfusion. Acute severe hypoxia typically results in cerebral vasodilatation and increased perfusion, [45] however, the activation of peripheral chemoreceptor activity triggered by hypoxia causes hyperventilation, leading to a decrease in CO₂ partial pressure and resulting in cerebral vasoconstriction [46]. The magnitude of the increase in brain perfusion depends on altitude and these opposing influences, [47] with hypoxia not substantially influencing CBF until arterial oxygen tension (PaO₂) drops below approximately 60 mmHg, corresponding to the steeper portion of the oxyhemoglobin dissociation curve. Lafave et al. (2019) demonstrated that participants crossed this threshold at 4240 m altitude, where PaO₂ decreased to 49.0 ± 5.2 mmHg and arterial oxygen saturation (SaO₂) fell to 85.2 ± 3.9% [49]. At this point, the vasodilatory effects of hypoxia overcame the vasoconstrictive effects of chronic hypocapnia (PaCO₂: 28.6 ± 3.4 mmHg), resulting in significant increases in global CBF from 699.7 ± 101.3 ml/min to 906.0 ± 204.3 ml/min. This compensatory cerebrovascular response is necessary to maintain adequate cerebral oxygen delivery despite reduced arterial oxygen content. Notably, while CBF increases significantly after ascent to high altitude, it typically normalises or returns to sea-level values after 1 week of acclimatisation [48].The extent of hypoxic exposure and individual cerebrovascular sensitivity to hypoxia and CO₂ are the main mechanisms underlying CBF regulation in such conditions, [47] representing a critical adaptive mechanism that helps preserve cerebral oxygenation during chronic hypobaric hypoxic exposure.

The classic study by Rahn and Otis [38] demonstrated that increased ventilation is a crucial mechanism for acclimatisation to high-altitude hypoxia. Their findings describe a diagonal shift to the right in the alveolar gas composition diagram, driven by a reduction in arterial CO_2_ (PaCO_2_) and an increase in arterial O_2_ (PaO_2_). In the acclimatised state, despite a lower PaO_2_, the body adapts by increasing ventilation and haematocrit to maintain O_2_ delivery at baseline levels. Increased ventilation reduces PaCO_2_, leading to mild respiratory alkalosis, which helps offset hypoxic vasodilatation. As a result, CBF and the cerebral metabolic rate of O_2_remain unchanged despite the hypoxic environment [50–52].

There is evidence that prolonged exposure to mild chronic hypoxia, such as that experienced at high altitudes, can lead to increased capillary density in the brain, at least in animal models like rats and mice [15, 53]. However, it remains uncertain whether this adaptation also occurs in humans living at high altitudes.

Surprisingly, the brain increases its use of fatty acids for energy during chronic hypoxia, although it typically relies on glucose and, during fasting, ketone bodies [54]. This metabolic shift toward fatty acid utilisation during mild chronic hypoxia may indicate an adaptive mechanism that improves O_2_ efficiency in the brain over time. Recent findings are consistent with evidence that marathon runners may experience a transient and reversible reduction in brain myelin content [55]. This is also consistent with rodent studies suggesting that lipids may serve as an energy reserve for neural cells [56]. 

Taken together, hypoxia studies suggest that neurons can survive much longer than previously believed, revealing a surprising resilience and greater potential for functional recovery than once assumed [57]. Current findings highlight the brain’s ability to maintain O_2_ homeostasis and energy production under conditions of mild chronic hypoxia and suggest the potential for structural remodelling driven by adaptive capacity at both the cellular and vascular levels.

**Fig. 1:**
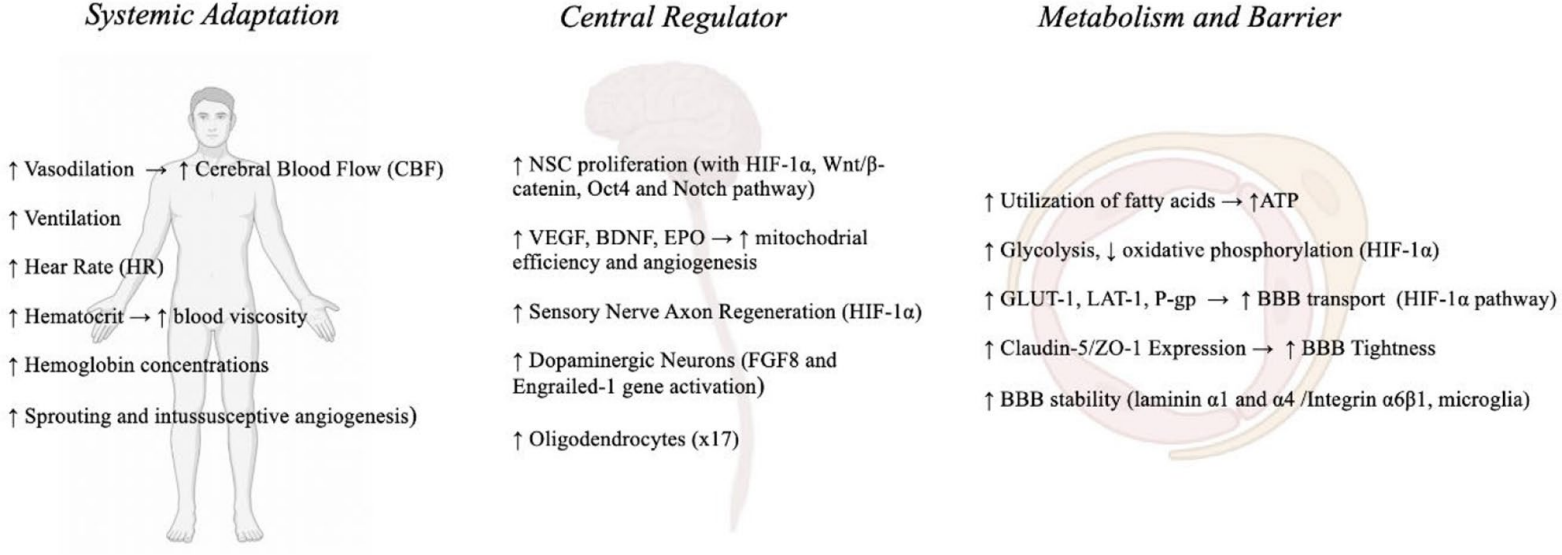
Multilevel adaptive and protective mechanisms activated in response to mild chronic hypoxia: systemic adjustments (increased cerebral blood flow and heart rate), cellular mechanisms (HIF-1α activation, metabolic shifts to glycolysis), neurotrophic support (VEGF, BDNF, EPO production), angiogenesis, neural stem cell activation, and blood-brain barrier modifications. These interconnected mechanisms work synergistically to maintain oxygen homeostasis and preserve brain function during mild chronic hypoxia. Cerebral blood flow (CBF), heart rate (HR), neural stem cell (NSC), hypoxia-inducible factor 1-alpha (HIF-1α), vascular endothelial growth factor (VEGF), brain-derived neurotrophic factor (BDNF), erythropoietin (EPO), adenosine triphosphate (ATP), glucose transporter 1 (GLUT-1), L-type amino acid transporter 1 (LAT-1), P-glycoprotein (P-gp), blood–brain barrier (BBB), zonula occludens-1 (ZO-1)

An overview of these key, multilevel adaptive mechanisms activated in response to hypoxia is presented in Fig. 1.

## Cellular models of mild chronic hypoxia

NSCs are multipotent neuronal precursor cells capable of self-renewal that, under specific physiological and pathological conditions, can differentiate into fully functional neurons, astrocytes, and oligodendrocytes during both development and adulthood [21]. The physiological conditions of mild hypoxia are particularly relevant because they strongly promote NSC self-renewal and improve their engraftment after transplantation into the brains of experimental models (typically 2.5–5% O₂ for 48–72 h in vitro).

Cultured NSCs constitute an important research model for analysing neurogenesis and the mechanisms that maintain neural cell complexity and plasticity. They are also the subject of intensive research focused on their potential use in therapies for brain injury and neurodegenerative diseases. Although NSCs play a crucial role in maintaining cellular homeostasis in the central nervous system (CNS) under physiological conditions, spontaneous regeneration after brain injury is limited, making the restoration of neuronal function possible primarily through exogenous cell transplantation. Available evidence suggests that transplanted NSCs support repair processes not only through direct cell replacement but also through alternative mechanisms, including neuroprotection, reduction of host cell death, stimulation of endogenous angiogenesis, modulation of the immune response during inflammatory damage, and elimination of neurotoxic molecules from the damaged tissue environment [27, 28]. 

 In vitro studies have demonstrated that culturing NSCs under mild hypoxic conditions significantly increases their proliferation and neurogenic potential. This is primarily attributed to the activation of key molecular pathways, including the HIF pathway, Wnt/β-catenin, Oct4, and Notch signalling, which are crucial for promoting self-renewal and cellular proliferation, thereby reducing apoptosis [58–60]. In contrast, exposure to severe hypoxia (less than 1% O_2_) strongly arrests the proliferation of stem cells, driving them into quiescence and even apoptosis, whereas standard normoxic conditions (20% O_2_) promote differentiation and mitotic arrest [29]. 

Under mild hypoxia, hypoxia-inducible factors (HIFs), particularly HIF-1α and HIF-2α, are stabilised and act as transcriptional regulators of genes involved in metabolism, angiogenesis, and the maintenance of stem cell properties [61]. Under normoxia, HIF-α is hydroxylated by prolyl hydroxylases (PHD), but during hypoxia, PHD activity is inhibited, leading to HIF-α stabilisation, nuclear translocation, dimerisation with hypoxia-inducible factor beta (HIF-β), and activation of target genes [61]. The main targets include components of the Notch pathway (HES1, HES5), pluripotency factors (c-Myc, Oct4, Sox2), and metabolic and vascular genes (*VEGF*,* EPO*,* GLUT1*). HIF-2α plays a central role in preserving the undifferentiated, self-renewing phenotype of NSCs. Hypoxia also enhances Notch signalling by upregulating Notch1 and its downstream effectors (e.g., HES1) through HIF-1α activity, maintaining NSCs in a quiescent and undifferentiated state while repressing the expression of transcription factors such as Mash1 and Neurogenin [27]. 

Moderate activation of the Wnt/β-catenin pathway supports NSC proliferation and self-renewal, with HIF-1α stabilising β-catenin and thereby increasing the expression of proliferative genes such as Cyclin D1 and pluripotency factors like Sox2 [62]. Hypoxia can transiently activate phosphoinositide 3-kinase (PI3K)/protein kinase B (Akt) signalling to enhance cell survival and proliferation, while chronic hypoxia suppresses mechanistic target of rapamycin (mTOR) activity, limiting differentiation and preserving the stem cell pool [63]. Furthermore, hypoxia modulates histone modifications and deoxyribonucleic acid (DNA) demethylation in the promoters of pluripotency genes (e.g., Oct4, Sox2), and HIFs recruit epigenetic enzymes such as histone demethylases JMJD1A and JMJD2B to activate stemness-related gene expression [64]. Finally, under hypoxia, NSCs shift from oxidative phosphorylation to glycolytic metabolism, reducing reactive oxygen species (ROS) production and protecting cells from oxidative stress and premature differentiation. HIF-1α induces the expression of glycolytic enzymes (lactate dehydrogenase A (LDHA), phosphofructokinase-1 (PFK1)) and glucose transporters (glucose transporter 1 (GLUT1) and glucose transporter 3 (GLUT3)), ensuring metabolic adaptation to low oxygen conditions [65]. Together, these mechanisms enable NSCs to maintain self-renewal, resist apoptosis, and preserve their differentiation potential under low-oxygen conditions.

Recent findings highlight the critical role of HIF-1α in facilitating axon regeneration in sensory neurons, particularly under conditions of mild chronic hypoxia (3–5% O₂ for 48 h). Sensory neurons, specifically those located in the dorsal root ganglia, exhibit a remarkable capacity for regeneration following peripheral nerve injuries - a phenomenon significantly enhanced by hypoxic conditions [22]. In cultured dorsal root ganglia neurons, exposure to mild chronic hypoxia has been shown to upregulate numerous injury-induced genes, with approximately 64% of known HIF-1α target genes activated following axonal injury [22]. This activation is crucial for initiating a pro-regenerative transcriptional programme that stimulates the intrinsic growth capacity of sensory neurons. Furthermore, studies demonstrate that the induction of HIF-1α through mild chronic hypoxia significantly enhances axon regeneration both in vitro and in vivo [22]. 

Importantly, mild hypoxia significantly impacts neuronal viability in rat cortical neurons via HIF-1α. During the first 24 h of hypoxia, HIF-1α is stabilised and translocated to the nucleus, regulating genes that promote cell survival (3–5% O₂, 24 h) [66]. After 24 h hypoxia leads to increased neuronal death, indicated by mitochondrial depolarisation, cytochrome c release, and caspase-3 activation. Knockdown of HIF-1α worsens these effects, highlighting its neuroprotective role early on. However, after 24 h, the protective effects of HIF-1α decline, with reduced expression and increased apoptosis [66]. Notably, overexpression of HIF-1α fails to enhance neuroprotection, indicating that the stabilised levels during mild hypoxia are sufficient for its functions and that exceeding these levels does not confer additional benefits.

Mild chronic hypoxia activates adaptive mechanisms that promote neuronal survival under conditions of mitochondrial dysfunction and oxidative stress (3–5% O₂ for 24–48 h). It stimulates the secretion of neuroprotective factors such as EPO, VEGF, and BDNF, which improve mitochondrial function, facilitate angiogenesis, and support NSC-mediated neurogenesis, thereby enhancing the overall metabolic environment for cell survival [28]. Research indicates that manipulating O_2_ levels in culture conditions can optimise the development of NSC-based therapies. Mild hypoxia not only preserves the stem-like properties of NSCs but also augments their therapeutic potential in the context of brain injuries and neurodegenerative diseases [27]. 

Mild chronic hypoxia also promotes the generation of tyrosine-hydroxylase-positive dopaminergic neurons from embryonic rat CNS precursors, which is particularly significant given the loss of this neuronal subtype in Parkinson’s disease [67]. Additionally, mild hypoxia supports the survival of tyrosine-hydroxylase-positive sympathoadrenal cells derived from neural crest stem cells and promotes the proliferation and differentiation of ventral midbrain precursors into dopaminergic neurons (5% O₂, 72 h) [20]. This substantial enhancement in dopaminergic neuron yield is accompanied by elevated expression of key genes such as FGF8 and engrailed-1, both known to promote dopaminergic differentiation [68]. Conversely, severe hypoxia represses neuronal differentiation [30]. 

In addition to promoting cell expansion, mild hypoxia significantly enhances the capacity of these precursors to generate oligodendrocytes. Specifically, precursors expanded at 5% O_2_ produce 17 times more oligodendrocytes upon differentiation than those cultured at 20% O_2_ [29]. Furthermore, when precursors initially expanded in 5% O_2_ are subsequently differentiated in 20% O_2_, oligodendrocyte maturation is further stimulated by an additional 2.5-fold. This indicates that the dynamic regulation of O_2_ tension is crucial for optimising both the proliferation and differentiation of CNS precursors, particularly in the context of oligodendrocyte lineage commitment [29]. The study reveals a novel interaction between O_2_ tension and bone morphogenetic protein signalling pathways. While bone morphogenetic protein signalling generally promotes astrocyte differentiation, mild hypoxia appears to repress this pathway, thereby allowing for greater preservation of precursor potential and enhanced oligodendrocyte generation [29]. In 5% O_2_, the cultures yielded a diverse range of differentiated cell types, including neurons, astrocytes, and oligodendrocytes, highlighting the multilineage competence promoted by mild chronic hypoxia. However, when cultured in 20% O_2_, differentiation was skewed predominantly toward the neuronal lineage, with a significant reduction in the generation of glial cells, particularly oligodendrocytes [23]. 

Mild chronic hypoxia has been identified as a potentially protective response in cellular models of mitochondrial dysfunction, particularly in the context of brain capillary endothelial cells, which are essential components of the BBB (5% O₂ for 48 h). Exposure to hypoxia resulted in the stabilisation and translocation of HIF-1α to the nucleus, which was associated with an upregulation of key BBB transport systems, including GLUT-1, LAT-1, and P-gp [69]. This upregulation was observed at both the transcriptomic and protein levels, indicating that hypoxia enhances the functional capabilities of brain capillary endothelial cells without compromising barrier integrity. Notably, the expression of tight junction proteins, including claudin-5 and ZO-1, remained stable, suggesting that hypoxia does not disrupt the physical barrier of the endothelial monolayer [69]. Hypoxic conditions led to a significant increase in transendothelial electrical resistance, indicating improved barrier tightness. These findings imply that mild chronic hypoxia (5% O₂ for 48 h) not only supports the maturation of brain endothelial cells but also promotes their functional efficacy, potentially contributing to improved cerebral health during conditions of mitochondrial dysfunction [70]. 

Glucose metabolism is critical in supporting the enhanced proliferation observed under hypoxic conditions. One study found that the stabilisation of HIF-1α during mild hypoxia promotes the expression of glycolytic enzymes and glucose transporters, facilitating a metabolic shift towards increased glycolysis [23]. This metabolic adaptation is vital for maintaining energy production and cellular function in an environment where mitochondrial oxidative phosphorylation may be compromised. Moreover, chronic hypoxia has been found to alter organ-specific metabolism in the brain, promoting fatty acid uptake and oxidation while enhancing glucose metabolism in certain contexts [54]. Studies have also evaluated the impact of O_2_ tension on the differentiation potential of neural precursors [23]. This metabolic flexibility is essential for maintaining cellular functions under varying O_2_ conditions and highlights the brain’s ability to adapt to chronically low-O_2_ environments. The findings indicate that mild chronic hypoxia improves the survival and expansion of neural precursor cells and modulates their metabolic pathways, promoting a reliance on glycolysis rather than oxidative phosphorylation.

Hence, mild chronic hypoxia promotes neuronal regeneration by increasing stem cell self-renewal, encouraging differentiation into neurons and glia, and stabilising brain endothelial function. Through HIF-1α-mediated pathways, it induces metabolic and transcriptional adaptations that enhance cellular resilience and survival under stress conditions. Consequently, the molecular and cellular responses to mild chronic hypoxia highlight its therapeutic potential in neurological disorders. By enhancing neurogenesis, promoting oligodendrocyte formation, and preserving BBB function, mild hypoxia may be harnessed to support regeneration and neuroprotection in diseases involving mitochondrial dysfunction, neurodegeneration, or trauma. Therefore, understanding the implications of mild chronic hypoxia in cellular models is essential for advancing regenerative medicine strategies and developing effective treatments for neurological disorders.

**Fig. 2:**
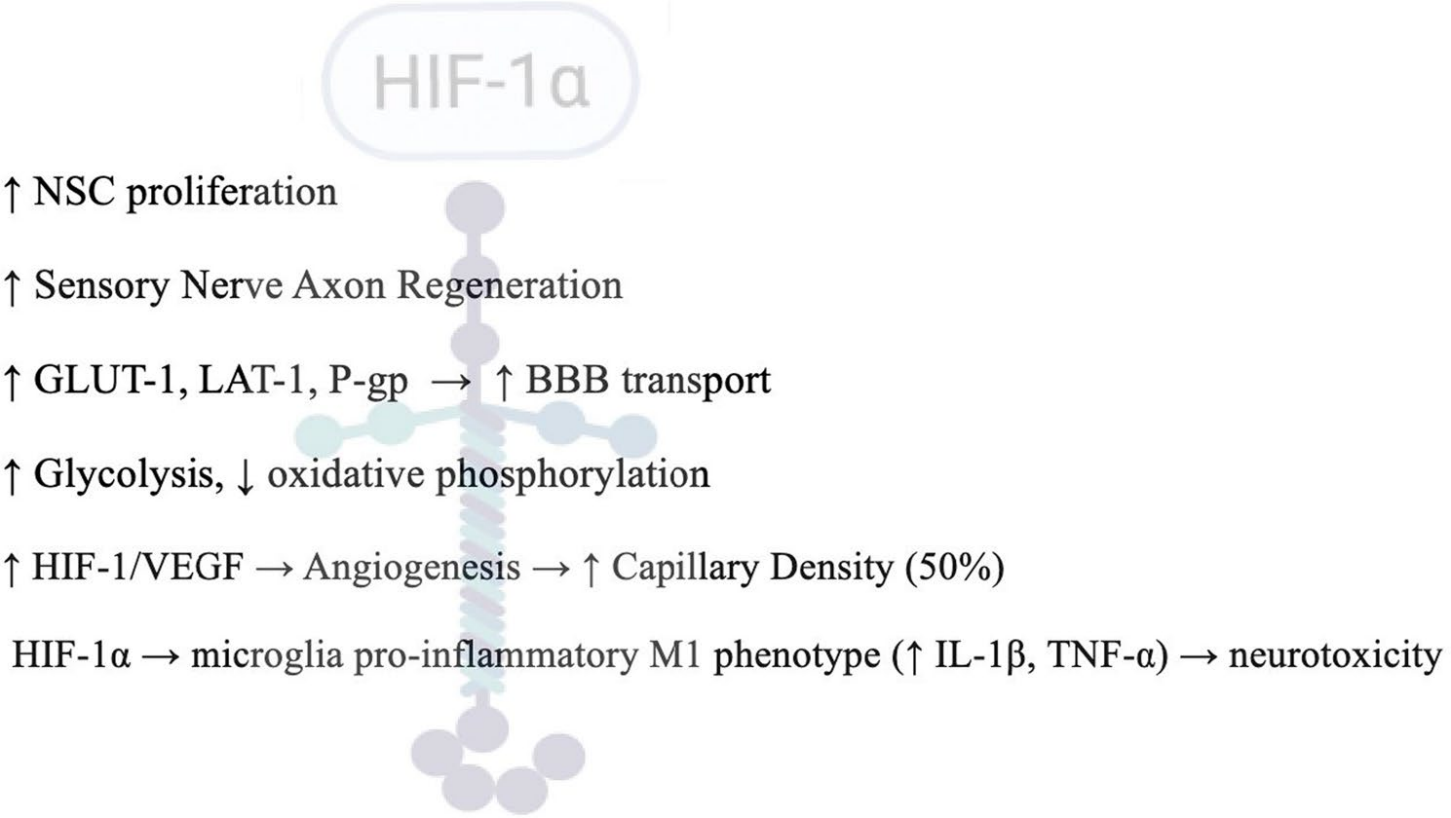
The effect of HIF-1α activation on adaptive metabolic, regenerative, and vascular processes in cells. HIF-1α acts as a master transcriptional regulator orchestrating cellular adaptation to mild hypoxia by promoting metabolic reprogramming (glycolytic shift), neural stem cell self-renewal, angiogenesis through VEGF upregulation, and blood-brain barrier transporter expression (GLUT1, LAT-1, P-gp) while maintaining barrier integrity. However, HIF-1α activation can also drive M1 pro-inflammatory microglial polarization, leading to increased production of pro-inflammatory cytokines (IL-1β, TNF-α) and potential neurotoxicity. Blood–brain barrier (BBB), hypoxia-inducible factor 1 alpha (HIF-1α), vascular endothelial growth factor (VEGF), neural stem cell (NSC), L-type amino acid transporter 1 (LAT-1), P-glycoprotein (P-gp), glucose transporter 1 (GLUT1), interleukin-1 beta (IL-1β), tumor necrosis factor alpha (TNF-α)

The activation of the HIF-1α constitutes a central mechanism in a range of adaptive changes. Figure 2. presents selected effects resulting from the activation of HIF-1α.

## Microvascular adaptations to mild chronic hypoxia

Mild chronic hypoxia triggers several microvascular adaptations to optimise O_2_ delivery and maintain tissue perfusion. These changes include structural remodelling, haemodynamic adjustments, and compensatory mechanisms, as evidenced by studies in animal models.

Physiological adaptation begins with an increase in haemoglobin concentration, leading to elevated PaO_2_ content despite low O_2_ tension. Haematocrit rises, increasing the O_2_-carrying capacity. This rise in haematocrit doubles blood viscosity, increasing shear stress on the vessel walls. This, in turn, triggers vasodilation via endothelial nitric oxide signalling, reducing peripheral resistance. Consequently, despite the increased viscosity, total peripheral resistance remains stable due to arteriolar dilatation and vascular remodelling. The heart rate decreases slightly to prevent hypertension, contributing to the maintenance of blood pressure through reduced cardiac output [71, 72]. It has been proposed that mild chronic normobaric hypoxia may combat hypertension in spontaneously hypertensive rats through VEGF-A-mediated angiogenesis, nitric oxide-dependent vasodilation, and renin–angiotensin system rebalancing, thereby addressing both vascular rarefaction and increased peripheral resistance [73]. 

Structural adaptations include sprouting and intussusceptive angiogenesis. New capillaries form to improve O_2_ diffusion, and existing vessels split to increase capillary density—a process well documented in the mouse brain and retina under mild chronic hypoxia [24, 72]. Exposure to chronic mild hypoxia promotes extensive remodelling of brain blood vessels. This brain-specific response is not observed in the vessels of the heart, skeletal muscles, kidneys, or liver. The result is a 50% increase in vascular density within 2 weeks [74]. 

LaManna et al. noted that the brain adapts to mild hypoxia through metabolic (both systemic and central) and vascular mechanisms triggered by HIF-1 [19]. The formation of brain vessels mediated by this factor is completed within 3 weeks of exposure and is reversible if normoxia is restored. Acclimatisation responses to hypoxia can be significantly impaired with ageing and in the presence of vascular and metabolic diseases [24, 75, 76]. Other researchers have found that although hypoxia-induced angiogenesis is dependent on HIF-1 transcriptional activation of VEGF, there are also HIF-1–independent mechanisms contributing to hypoxia-induced angiogenesis in the ageing rat brain [77, 78]. 

Hypoxia-induced angiogenesis in the brain can occur through several HIF-independent pathways. Under low-oxygen conditions, the unfolded protein response (UPR) can be activated in endothelial cells, leading to upregulation of pro-angiogenic factors such as VEGF via activating transcription factor 4 (ATF4) and x-box binding protein 1 (XBP1) signalling, independent of HIF stabilization [79, 80]. Additionally, hypoxia can trigger the production of ROS that activate redox-sensitive transcription factors like nuclear factor kappa B (NF-κB) and activator protein 1 (AP-1) which directly induce expression of angiogenic genes, including interleukin-8 (IL-8) and angiopoietin-2 [81, 82]. The mTOR pathway also contributes to HIF-independent angiogenesis by regulating translation of VEGF messenger ribonucleic acid (mRNA) through its effects on ribosomal proteins and translation initiation factors [83]. Furthermore, hypoxia can modulate microRNA (miRNA) expression, particularly downregulation of anti-angiogenic miRNAs like miR-15 and miR-16, through mechanisms involving histone modifications and DNA methylation changes that occur independently of HIF transcriptional activity [84, 85]. Notch signaling, which is critical for arterial specification and vascular patterning in the brain, can also be modulated by oxygen tension through HIF-independent mechanisms involving direct effects on γ-secretase activity and Delta-like ligand expression [86, 87]. These parallel pathways ensure robust angiogenic responses even when HIF signalling is compromised, highlighting the redundancy and complexity of oxygen-sensing mechanisms in cerebral vasculature.

Halder et al. found that chronic mild hypoxia (8% O_2_ for 2 weeks in mice) caused endothelial proliferation and extravascular fibrinogen leakage predominantly in capillaries and, to a lesser extent, in venules [88]. Importantly, endothelial proliferation and extravascular fibrinogen leakage did not occur in the same locations, suggesting that the leakage in non-angiogenic vessels represents a dysfunctional response to hypoxia rather than a side effect of new vessel formation. Endothelial proliferation was linked to intravascular fibrinogen deposits in unstable blood vessels. Chronic mild hypoxia induced transient vascular leakage, particularly in the olfactory bulb and brainstem, accompanied by marked aggregation and activation of microglia. Depletion of microglia led to greater cerebrovascular leakage during hypoxia, and this leakage was associated with higher endothelial cells MECA-32 immunoreactivity and a reduction in the tight junction proteins ZO-1 and occludin in endothelial cells. MECA-32 is a monoclonal antibody commonly used as a marker for murine endothelial cells. Endothelial proliferation was also significantly higher in brain areas more susceptible to barrier permeability [89]. 

Furthermore, chronic mild hypoxia (8% O_2_ for 2 weeks in mice) induced extensive vascular remodelling in the brain, including endothelial proliferation, angiogenesis, and increased expression of tight junction proteins, suggesting enhanced vascular stability. Halder et al. observed a substantial upregulation of laminin α1 and α4 subunits in cerebral blood vessels, associated with increased expression of laminin 111 and 411, while the α2 and α5 subunits remained unchanged [3]. Additionally, there was significant endothelial upregulation of the laminin receptor integrin α6β1, which was concluded to augment BBB stability under mild hypoxia [3]. Sapkota et al. demonstrated that chronic mild hypoxia triggered vascular remodelling in both young and old mouse brains, affecting the expression of cerebral vascular integrin β4 and its ligands, laminin 411 and 511 (α4 and α5 subunits). They reported that integrin β4 expression differs between white and grey matter capillaries, suggesting region-specific regulation of the vascular response to hypoxia [90]. 

Taken together, these findings indicate that chronic mild hypoxia induces distinct responses in proliferating and non-proliferating vessels. Angiogenic vessels exhibit endothelial proliferation and transient fibrinogen deposition, whereas non-angiogenic vessels display extravascular fibrinogen leakage. Importantly, endothelial proliferation and vascular leakage never co-localise, suggesting that extravascular leakage is not a side effect of angiogenic endothelial proliferation but rather a dysfunctional response to hypoxia unique to mature vessels.

## Direct effects of chronic mild hypoxia on the BBB, BSCB, and BCSFB

The BBB protects the brain by selectively allowing the passage of essential substances from the blood while blocking harmful ones, thereby maintaining a stable brain environment and supporting neurological health. The BBB is formed by inter-endothelial tight junctions, a vascular basal lamina, and associated structures such as extracellular matrix proteins, astrocyte end-feet, and pericytes [91–93]. Similarly, the BSCB protects the central nervous system and shares a structural similarity with the BBB. However, the BSCB exhibits relatively higher permeability due to reduced levels of specific tight junction proteins, such as occludin and ZO-1. The BSCB also differs from the BBB in its local immune responses and its support from vertebral disc tissues, which influence both healthy neural tissue and the development of pathophysiological processes in the spinal cord [94, 95]. The BCSFB is a highly selective interface located primarily at the choroid plexus epithelium that separates the blood from the CSF in the brain’s ventricular system. It regulates the exchange of substances between the bloodstream and the CSF, maintaining the brain’s extracellular fluid homeostasis [96–98]. 

The role of integrin β1 in BBB integrity during normoxia and hypoxia was investigated by Halder et al., who showed that it is essential for maintaining BBB integrity and for mediating vascular remodelling induced by mild hypoxia [99]. Blocking integrin β1 significantly affected the BBB of young mice, making it resemble that of aged mice, suggesting that enhancing integrin β1 function might help limit the effects of ageing on the BBB. Burnier et al. found that activated protein C is crucial for cerebral blood vessel remodelling in response to chronic mild hypoxia because its blockade abolished endothelial proliferation and the upregulation of integrins α5β1 and αvβ3. [100]

Tight junctions, including claudins (especially claudin-5), occludin, and ZO-1, are vital for maintaining BBB integrity and regulating paracellular permeability [74, 101–105]. The increased BBB disruption observed in aged mice under chronic mild hypoxia may be due to a delayed vascular remodelling response and reduced vascular protection provided by microglia [4]. Vascular permeability in the spinal cord also increases with age, although ageing did not significantly affect endothelial proliferation. While microglial activation was higher in aged mice under hypoxia, the vasculoprotective function of microglia declined with age. Vascular disruption in the spinal cord was linked to the loss of white matter myelin and oligodendrocytes in older mice [106]. Halder et al. further highlighted the essential role of microglia in supporting BSCB integrity during hypoxia, observing transient vascular leakage and microglial clustering. Microglial depletion increased vascular leakage, and a peptide blocking fibrinogen binding to its Mac-1 integrin receptor diminished the microglial protective effect, suggesting a fibrinogen–Mac-1 interaction as a potential mechanism [107]. 

However, the role of microglia in BSCB regulation extends beyond this single pathway. Haruwaka et al. showed that microglia exhibit dual effects on barrier permeability during systemic inflammation, with certain microglial subpopulations protecting vascular integrity while others promote barrier disruption, demonstrating the ambiguous nature of microglial-vascular interactions [108]. Furthermore, Akiyoshi et al. found that microglia actively enhance synaptic activity and promote local network synchronization in the CNS, suggesting that their role in the spinal cord encompasses both vascular support and neuronal circuit modulation [109]. With regard to hypoxia-induced BSCB dysfunction, microglia not only regulate tight junction protein expression but also modulate the inflammatory microenvironment through the release of cytokines such as TNF-α and IL-1β, which can either stabilize or compromise barrier integrity depending on the duration and severity of hypoxic exposure [107, 108]. The spatial distribution of microglia along the BSCB differs from that at the BBB, with higher densities observed in spinal cord white matter, where they provide enhanced surveillance and protection against hypoxia-induced vascular damage [99, 106]. This regional specialization of microglial function may explain the differential susceptibility of white and grey matter vessels to hypoxic injury in the spinal cord.

Halder et al. found that chronic mild hypoxia (8% O_2_ for up to 1 week) promoted endothelial proliferation and increased vascularity, elevated fibronectin expression, and increased endothelial expression of integrin α5β1 in the spinal cord [110]. Tight junction proteins (claudin-5, ZO-1, and occludin) were upregulated, and astrocytes were activated. Mice deficient in integrin α5 showed reduced vascular remodelling in the spinal cord, indicating a significant role for integrin α5β1 in supporting endothelial proliferation. Integrin β1 is also critical for maintaining spinal cord vessel integrity, with evidence suggesting a difference in dependence on integrin β1 function during hypoxia between white and grey matter vessels, the white matter being more susceptible [99]. 

Barakat et al. demonstrated that exposure to 8% O₂ for up to 48 h in rats led to ~ 30% cell death in the choroid plexus and ependymal layer, along with significant damage to the choroid plexus epithelium. Apoptotic changes were evident in choroid plexus cells, including disrupted microvilli, mitochondrial loss, chromatin condensation abnormalities, and cytoplasmic vacuolation. [1, 111] This damage may significantly impair CSF production and flow. Initially, during the first 24 h, choroid plexus cells were more resilient than ependymal layer cells, likely due to the abundant blood supply within the choroid plexus and the resulting availability of glucose and other substrates for glycolysis. However, by 48 h, approximately one-third of the cells in both structures had died, and further damage occurred during the recovery period [1]. 

Severe hypoxemia induced by exposure of rats to 8% oxygen also increases protein concentration in the CSF, with a significant rise in the albumin-to-total protein ratio after just 6 h, indicating that BCSFB disruption is the main driver of this effect [1]. Moreover, these negative effects are modulated by hypoxia-related cytokines: of 32 hypoxia- and inflammation-associated cytokines measured in the CSF, 12 decreased significantly (including follistatin-like 1 (FSTL1), interferon-γ-inducible protein 10 (IP10), secreted protein acidic and rich in cysteine (SPARC), interleukin-17 A (IL-17 A), interleukin-15 (IL-15), TNFα, IL-6, granulocyte–macrophage colony-stimulating factor (GM-CSF), leukemia inhibitory factor (LIF), fibroblast growth factor 21 (FGF21), granulocyte colony-stimulating factor (G-CSF), and myostatin/growth differentiation factor 8 (MSTN/GDF8)), while EPO and VEGF increased markedly [1]. 

In contrast to this selective cytokine modulation observed during short-term severe hypoxemia, prolonged systemic exposure to 10% O₂ has been shown to trigger a robust inflammatory response. Mesentier-Louro et al. demonstrated that 7 days of systemic hypoxia at 10% O₂ induced a significant increase in ten pro-inflammatory cytokines in plasma and four in retinal homogenates within one hour post-exposure, including IL-6, IL-13, IL-1β, VEGF, and IL-22, accompanied by upregulation of monocyte chemotactic protein-3 (MCP-3) [16].These findings indicate that systemic hypoxia of moderate-to-severe intensity provokes an early, transient but intense inflammatory reaction involving both systemic and CNS compartments. The simultaneous rise in IL-1β and VEGF levels in plasma and retina suggests a close interplay between vascular permeability, glial activation, and neuroinflammation. Additionally, increased expression of aquaporin-4 (AQP-4) and downregulation of Kir4.1 in retinal glial cells were observed, consistent with disturbed osmotic balance and post-hypoxic retinal oedema [16]. This glial reactivity and cytokine surge reflect a broader CNS response to systemic hypoxia, in which the early inflammatory phase may precede oedema formation and subsequent neurodegeneration.

**Fig. 3:**
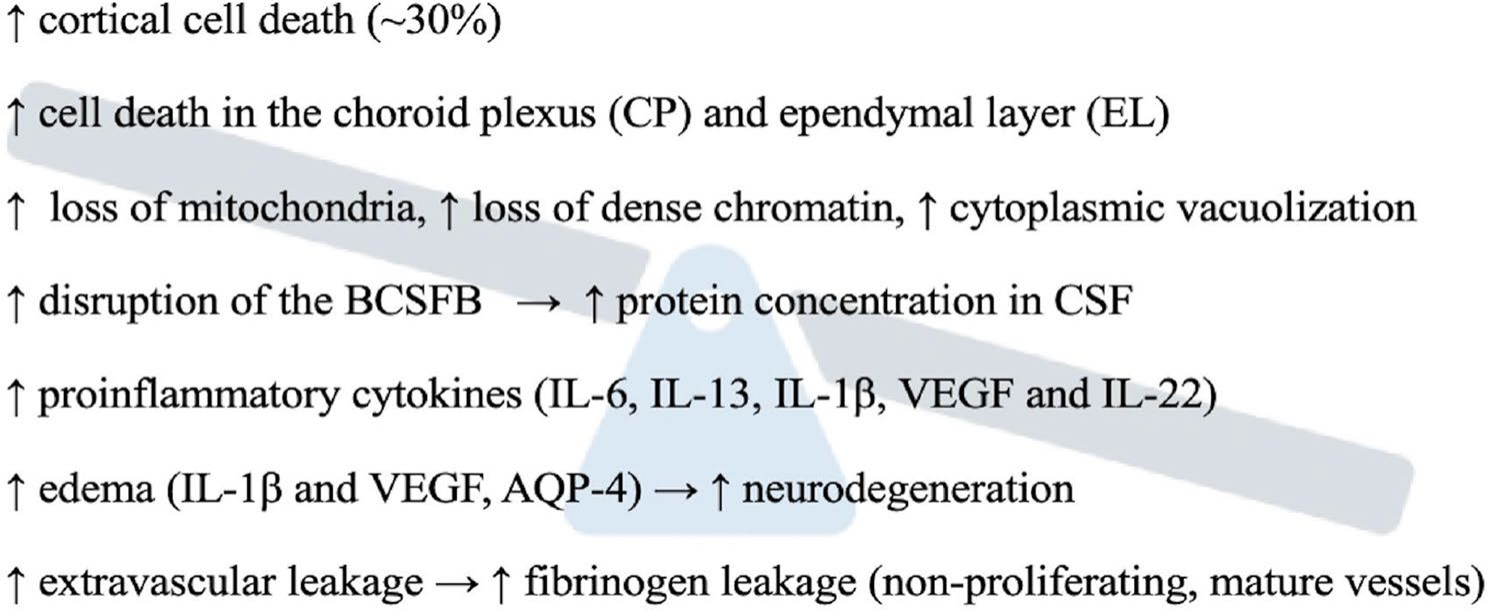
Cellular damage and inflammation due to severe hypoxemia evoked by 8–10% O_2_ Severe hypoxemia causes destructive effects on brain barrier systems, including choroid plexus and ependymal layer damage (~ 30% cell death within 48 h), blood-cerebrospinal fluid barrier disruption with increased CSF protein content (elevated albumin-to-total protein ratio), and a robust pro-inflammatory cascade. The inflammatory response involves elevated pro-inflammatory cytokines (IL-6, IL-13, IL-1β, IL-22) and VEGF in both plasma and CNS compartments, accompanied by glial activation with increased AQP-4 expression, disrupted CSF homeostasis, and osmotic imbalance that precedes oedema formation and neurodegeneration. Choroid plexus (CP), ependymal layer (EL), blood-cerebrospinal fluid barrier (BCSFB), cerebrospinal fluid (CSF), interleukin-6 (IL-6), interleukin-13 (IL-13), interleukin-1 beta (IL-1β), vascular endothelial growth factor (VEGF), interleukin-22 (IL-22), interleukin-1 beta (IL-1β), aquaporin-4 (AQP-4)

A summary of the key destructive cellular and molecular changes, which are a direct consequence of severe hypoxemia, is presented in Fig. 3.

In conclusion, studies in experimental animal models have shown that the pathophysiology of mild chronic hypoxia on the BBB and BSCB is complex, involving both the direct effects of mild hypoxia on the brain and spinal cord and the contribution of systemic inflammation. Mild chronic hypoxia induces various changes in proteins responsible for the maintenance of BBB and BSCB integrity, including laminins, integrins, claudins, and ZO-1. These changes can differ between the brain and spinal cord and are modulated by factors such as age. Microglia play context-dependent roles in maintaining barrier integrity in the CNS. During systemic inflammation, microglia exhibit dual effects: initially protective through Claudin-5 expression and endothelial contact, but potentially disruptive during sustained inflammation through phagocytosis of astrocytic end-feet. Acute severe hypoxemia can rapidly compromise BCSFB cellular integrity and CSF homeostasis, triggering substantial pro-inflammatory cytokine release, glial activation, and structural disruption. This early inflammatory phase may precede oedema formation and subsequent neurodegeneration.

## The dual role of mild chronic hypoxia in respiratory virus-induced neuroinflammation

Hypoxic exposures of various intensities induced by respiratory viral infections represent a clear example of hypoxia’s dual role in the pathogenesis of neurological complications, functioning as both a trigger of immune dysregulation and a facilitator of BBB compromise [112, 113]. Understanding the interplay between HIF-1α-mediated immune activation and BBB disruption is critical, as these processes create a vicious cycle where hypoxia and inflammation continuously reinforce each other.

HIF-1α acts as a master transcriptional regulator that directly modulates immune cell phenotypes and inflammatory responses in both the periphery and the CNS [114]. In microglial cells, HIF-1α activation has been directly associated with polarization toward the M1 pro-inflammatory phenotype, characterized by increased expression of inducible nitric oxide synthase (iNOS) and enhanced secretion of pro-inflammatory cytokines including interleukin IL-1β, IL-6, and TNF-α [115–117]. This, in turn, promotes ROS production and creates a neurotoxic microenvironment that facilitates neuronal damage. Studies with human respiratory syncytial virus (hRSV) have demonstrated that the virus drives M1 microglial activation, suggesting a causal relationship between HIF-1α-mediated microglial activation and neuronal dysfunction [116]. 

Furthermore, HIF-1α acts as a transcription factor that directly upregulates the expression of pro-inflammatory cytokine genes [112, 118]. In influenza A infections, HIF-1α activation in lung epithelial cells has been shown to drive viral replication and cytokine production through signalling via the PI3K, mitogen-activated protein kinase (MAPK), and NF-κB pathways [118]. The same pathways are active in CNS resident cells, suggesting that systemic hypoxia can trigger a parallel systemic inflammatory cascade and neuroinflammation.

Importantly, the relationship between viral infection and HIF-1α activation does not always correlate directly with oxygen availability. Studies with hRSV have revealed that HIF-1α activation occurs independently of oxygen variations, indicating that viral pathogen-associated molecular patterns (PAMPs) or damage-associated molecular patterns (DAMPs) may trigger HIF-1α stabilization through non-canonical pathways [119]. This virus-induced HIF-1α stabilization may explain the neurological manifestations observed even in patients without overt respiratory failure, as the inflammatory signalling proceeds regardless of actual tissue oxygen exposure [112, 120]. Notably, HIF-1α can also have inhibitory effects on viral replication in certain situations, as demonstrated by studies showing that HIF-1α downregulates nucleolin expression, an hRSV receptor, potentially limiting viral spread [121]. Conversely, HIF-1α deficiency has been shown to enhance influenza A virus replication by promoting autophagy in alveolar epithelial cells [122], highlighting the complexity of HIF-1α’s role in viral infection pathophysiology.

Apart from its direct effects on CNS-resident immune cells, hypoxia functions as an indirect facilitator of neuroinflammation by compromising the structural and functional integrity of the BBB [123, 124]. The BBB, serves as a critical interface that restricts the entry of peripheral immune cells and inflammatory mediators into the CNS parenchyma [125]. Hypoxia destabilizes this barrier through multiple complementary mechanisms.

In vitro models of BBB disruption have demonstrated that hypoxia reduces the expression of tight junction proteins, particularly ZO-1 and claudin-5 [123, 124, 126]. This molecular disassembly of inter-endothelial junctions increases paracellular permeability, allowing the passage of peripheral macromolecules and immune cells into the CNS. Studies with severe acute respiratory syndrome coronavirus 2 (SARS-CoV-2) have shown that viral infection leads to increased BBB permeability, evidenced by Evans blue dye extravasation in animal models, accompanied by elevated levels of matrix metalloproteinase 9 (MMP9), which actively degrades collagen IV and basement membrane components [127]. Importantly, while tight junction integrity may be preserved in some cases, basement membrane disruption still facilitates viral invasion, suggesting a transcellular pathway for BBB crossing [127]. 

The astrocytic contribution to BBB maintenance is particularly vulnerable to hypoxic stress. Astrocytes, which regulate BBB formation and maintenance through the secretion of trophic factors and the expression of polarized transporters, undergo reactive changes in response to hypoxia [31, 32]. Following hRSV infection, increased glial fibrillary acidic protein (GFAP) expression and astrocyte activation coincide with BBB disruption at 60 days post-infection, suggesting that prolonged astrocytic reactivity perpetuates barrier dysfunction [128]. The hypoxia-induced astrocytic phenotype is characterized by altered metabolic profiles, including differential expression of proteins associated with glycolysis and gluconeogenesis, which may compromise their BBB-supporting functions [129, 130]. Studies using brain organoids have demonstrated that SARS-CoV-2 infection of astrocytes via the neuropilin-1 (NRP-1) receptor induces type I interferon pathway activation and proinflammatory cytokine production, leading to neuronal dysfunction and death even without direct neuronal infection [129, 130]. 

Studies with influenza A virus have demonstrated that infection promotes microglial activation alongside changes in tight junction protein expression, accompanied by the infiltration of CD45high CD11b + infiltrating macrophages/monocytes (CD45hi CD11b+), CD45high CD11b − infiltrating lymphocytes (CD45hi CD11b−) peripheral immune cells into the brain parenchyma [131]. This peripheral immune cell infiltration augments the neuroinflammatory response initiated by resident microglia and astrocytes, creating a self-perpetuating cycle of inflammation. Type I and II interferons, whose expression is upregulated in both microglia and infiltrating immune cells, have been specifically associated with facilitating immune cell entry through the BBB [131, 132]. 

The convergence of direct HIF-1α-mediated immune activation and indirect BBB compromise creates a synergistic vicious circle of neuroinflammation [112, 133, 134]. Once the BBB permeability is increased, systemically circulating cytokines, including those produced in the inflamed lung tissue, gain access to the CNS, where they encounter HIF-1α-primed microglia and astrocytes that are poised for exaggerated inflammatory responses [135, 136]. This bidirectional communication between the periphery and the CNS establishes a pathological feedback loop.

**Fig. 4:**
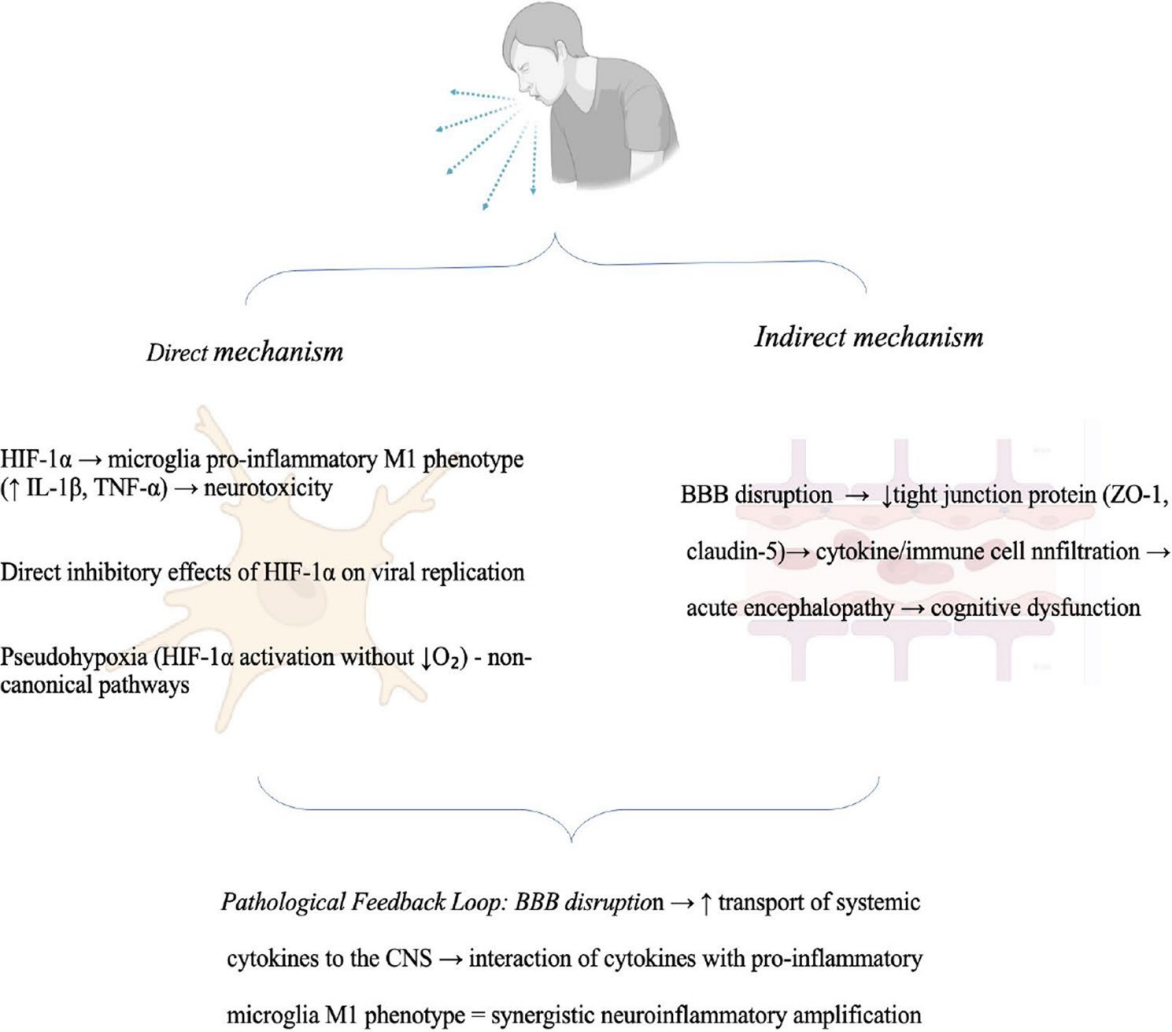
The effect of systemic infection on the CNS via a direct mechanism (HIF-1α/microglia) and an indirect mechanism (BBB disruption). Systemic infections cause neurological complications through dual pathways: a direct mechanism where HIF-1α activation (which can occur independently of oxygen levels via viral PAMPs or DAMPs) drives M1 pro-inflammatory microglial polarization and production of neurotoxic cytokines (IL-1β, TNF-α), creating a neurotoxic microenvironment; and an indirect mechanism where hypoxia compromises blood-brain barrier integrity by reducing tight junction protein expression (ZO-1, claudin-5), increasing paracellular permeability and allowing peripheral immune cell infiltration. These two mechanisms create a self-perpetuating vicious cycle where hypoxia-primed CNS immune cells encounter systemic inflammatory mediators crossing the compromised barrier, continuously amplifying neuroinflammation. Blood–brain barrier (BBB), central nervous system (CNS), hypoxia-inducible factor 1-alpha (HIF-1α), interleukin-1 beta (IL-1β), tumor necrosis factor alpha (TNF-α), zonula occludens-1 (ZO-1)

In SARS-CoV-2 infection, this dual mechanism is particularly evident (Fig. 4). The virus induces systemic hypoxia that stabilizes HIF-1α in both peripheral and central immune cells, while simultaneously compromising the BBB through direct viral infection of endothelial cells and pericytes [113, 115, 127]. Post-mortem analyses of COVID-19 patients have revealed microgliosis, astrogliosis, and evidence of hypoxia-related injuries in the cerebellum and cerebrum, alongside markers of BBB disruption [137, 138]. Studies in non-human primates have shown that SARS-CoV-2 infection leads to neuroinflammation associated with brain hypoxia, with viral RNA detected in brain tissue and colocalization of viral proteins with neuronal and glial markers [137, 138]. The clinical manifestation of “silent hypoxia” in COVID-19 patients, where severe hypoxemia occurs without dyspnea, has been hypothesized to result from direct CNS involvement, potentially through hypoxia-induced neuronal damage in respiratory control centres [120]. 

The temporal dynamics of these processes may determine the spectrum of neurological outcomes. Acute BBB disruption allows rapid infiltration of peripheral immune cells and inflammatory mediators, potentially contributing to acute encephalopathy and seizures observed during active infection [126, 128]. Conversely, chronic HIF-1α-mediated glial activation, even in the absence of persistent hypoxia, may underlie the long-term neuropsychiatric sequelae, including cognitive impairment, depression, and anxiety disorders reported in post-viral syndromes [120, 136, 139]. In SARS-CoV-2 infection, activation of the NLR family pyrin domain containing 3 (NLRP3) inflammasome in microglia through the spike protein contributes to sustained neuroinflammation and may play a role in the persistent cognitive deficits observed in long-term COVID [140]. Furthermore, SARS-CoV-2 infection has been shown to promote microglial-mediated synapse elimination in human brain organoids, with upregulation of genes associated with interferon signalling, phagocytosis, and synaptic pruning, providing a potential mechanistic explanation for the cognitive dysfunction seen in affected patients [132]. 

Understanding hypoxia’s dual role in neuroinflammation has important therapeutic implications [141]. Interventions targeting HIF-1α signalling may interrupt the direct inflammatory activation of glial cells, while strategies aimed at preserving BBB integrity could prevent the infiltration of peripheral immune cells into the CNS [114, 124]. The observation that HIF-1α can be activated through non-canonical, oxygen-independent pathways suggests that oxygen therapy alone may be insufficient to prevent neurological complications, necessitating more targeted anti-inflammatory approaches [118, 119]. 

Furthermore, the recognition that different respiratory viruses interact distinctly with the HIF-1α pathway (with some viruses stabilizing HIF-1α for replication while others are inhibited by it) underscores the need for virus-specific therapeutic strategies [121, 122, 142]. The potential for pharmacological modulation of microglial polarization, combined with BBB-protective strategies such as matrix metalloproteinase (MMP) inhibition or astrocytic support, represents a promising avenue for preventing and treating respiratory virus-induced neurological complications [117, 127, 143]. Targeting specific pathways such as toll-like receptor 2 (TLR2) - mediated neuroinflammation, which has been implicated in SARS-CoV-2-induced depression-like behaviours, may offer additional therapeutic opportunities [126]. 

Hypoxia serves as both a direct trigger of CNS inflammation through HIF-1α-mediated immune cell activation and an indirect facilitator through BBB compromise and peripheral immune cell infiltration [112, 133]. This dual mechanism explains the diverse neurological manifestations observed during and after respiratory viral infections, ranging from acute encephalopathy to chronic neurocognitive impairment [136, 139]. Future research should focus on dissecting the relative contributions of these direct and indirect pathways in different viral infections, identifying biomarkers for BBB integrity during acute illness, and developing targeted interventions that address both arms of this pathological cascade. Such integrative approaches hold promise for reducing the substantial neurological burden associated with respiratory viral infections.

## Neuroprotective properties of combined hypoxia-hypercapnia and roles of acidosis and microcirculation disorders

While this review has focused on the effects of isolated mild chronic hypoxia, it is important to recognize that in real clinical and physiological conditions, hypoxia rarely occurs in isolation. Rather, hypoxia is frequently accompanied by hypercapnia, acidosis, and microcirculatory disturbances, which profoundly modify the final outcomes. This section addresses these combined conditions and their clinical relevance, including the emerging therapeutic potential of combined hypoxia-hypercapnia exposure and the pathological role of acidosis and microcirculatory dysfunction.

Experimental evidence demonstrates that the combination of hypoxia and hypercapnia (hypercapnic hypoxia) provides significantly greater neuroprotective effects than either condition alone. In a landmark study by Tregub et al., [144] rats subjected to combined hypercapnia (PaCO₂ = 50 mmHg) and normobaric hypoxia (PaO₂ = 90 mmHg) showed superior protection against focal cerebral ischemic injury compared to isolated exposure to either condition. The combined exposure resulted in the smallest infarction volumes and most efficient reduction in neurologic deficits, whereas normobaric hypoxia alone had no significant impact in this experimental model.

The mechanisms underlying such synergistic neuroprotection are multifactorial and involve differential contributions from hypoxic and hypercapnic components. While hypoxia primarily activates HIF-1α and adenosine A1 receptor pathways [145], hypercapnia exerts dominant effects on NF-κB signalling, antioxidant systems, apoptosis inhibition, and maintenance of BBB integrity [146]. Zhou et al. [147] demonstrated that permissive hypercapnia has protective potential after global cerebral ischemia, with optimal effects at PaCO₂ levels of 60–100 mmHg [148]. Notably, experimental studies consistently indicate that hypercapnia is the predominant factor in the neuroprotective effect of the combined exposure [149]. 

At the cellular level, combined hypoxia-hypercapnia exposure produces pronounced ultrastructural changes in neurons, including hypertrophy of mitochondria and expansion of granular endoplasmic reticulum compartments, both indicators of enhanced metabolic capacity [50]. Studies using both in vivo and in vitro models demonstrate that hypercapnia and hypoxia stimulate proliferation of astrocytes and neurons [150] and result in inhibition of both caspase-dependent and caspase-independent apoptotic pathways [146]. Importantly, combined hypercapnic-hypoxic training achieves protective efficacy after as few as three exposures, considerably fewer than the seven or more sessions typically required for hypoxic training alone [151], suggesting enhanced therapeutic efficiency.

Recent comprehensive reviews have emphasized that hypercapnia not only acts as a potent neuroprotector but also increases tissue tolerance to ischemia and reperfusion through modulation of energy metabolism, activation of adaptive signalling pathways, reduction of cellular damage, regulation of pro-inflammatory factors, and activation of angiogenesis and neuronal processes [152]. The relationship between hypoxia-hypercapnia tolerance and life expectancy has also been explored, suggesting broader implications for aging and longevity research [153]. 

The relationship between hypoxia, acidosis, and neuronal damage represents a critical but often under-addressed aspect of cerebral hypoxic injury. During severe ischemia and tissue hypoxia, the shift to anaerobic glycolysis leads to substantial lactic acid accumulation. Under conditions of impaired perfusion, tissue lactate concentrations can exceed 20–25 µmol/g, causing extracellular pH to fall to approximately 6.0 [154]. Such degree of lactic acidosis severely hampers metabolic and functional recovery upon reoxygenation and is associated with irreversible cellular damage [154, 155]. The phenomenon was extensively characterized by Siesjö and colleagues, who demonstrated that lactic acidosis is an important component of the pathogenetic events leading to ischemic brain damage [156]. 

It is crucial to distinguish between respiratory acidosis (from elevated CO₂) and metabolic acidosis (from lactate accumulation), as their effects on neural tissue differ substantially. Severe hypercapnia (arterial PaCO₂ ~300 mmHg) can reduce tissue pH to approximately 6.6 without causing deterioration of the cerebral energy utilisation or morphological evidence of irreversible cell damage [154]. In contrast, lactic acidosis at equivalent pH levels is considerably more neurotoxic. This differential toxicity likely results from the rapid transmembrane transit of the neutral protonated form of lactic acid, which depletes intracellular buffer stores more rapidly than CO₂-mediated acidosis [157]. In vitro studies demonstrate that neurons can survive brief exposures to HCl-acidified media at pH 3.8, whereas lactic acidosis at pH 4.9 is lethal to over half of neurons and glia [157]. 

The pathophysiological mechanisms by which acidosis contributes to neuronal injury are diverse and include: (1) increased permeability of acid-sensing ion channels leading to pathological calcium influx [147, 158], (2) contribution to oxidative and excitotoxic injury [147], (3) impairment of synaptic transmission and long-term potentiation [159], (4) reduced astrocytic glutamate uptake capacity [147], (5) disruption of BBB integrity [147], and (6) effects on mitochondrial function through increased opening of the permeability transition pore [147]. Enhanced lipid peroxidation and protein denaturation have also been documented under lactic acidosis [160]. 

Clinical studies in traumatic brain injury patients have demonstrated that acidosis in the absence of hypoxia, characterized by elevated lactate and lactate/pyruvate ratios with normal tissue oxygenation, represents a distinct pathophysiology associated with the highest mortality [161]. This clinical observation underscores that acidosis itself, independent of hypoxia, constitutes a major pathological mechanism. Preischemic hyperglycemia aggravates ischemic brain injury through enhancement of lactic acidosis, with intracellular pH falling to 5.95 in hyperglycemic animals compared to 6.35 in normoglycemic controls during ischemia [156]. Studies on chronic hypoxia in neuronal cell cultures confirm that metabolic acidosis develops rapidly during hypoxic insults, with elevated medium lactate and depressed pH preceding neuronal dysfunction [132]. 

Interestingly, some in vitro studies suggest that the adverse effects of lactic acidosis observed in vivo may partly reflect vascular rather than direct neuronal toxicity. Schurr et al. [162] demonstrated that hippocampal slices could tolerate moderate lactic acid concentrations during hypoxia, suggesting neurons possess substantial intrinsic buffering capacity. This finding implies that the cerebrovascular system may be particularly vulnerable to lactic acidosis, contributing to perfusion deficits with the secondary neuronal damage.

Microcirculatory dysfunction represents both a cause and consequence of hypoxic brain injury, creating vicious cycles that lead to augmented tissue damage. During hypoxia, the cerebral microcirculation undergoes complex regional adaptations that are highly heterogeneous. Studies in hypoxic rats (7% O₂) demonstrate that cerebral capillary blood flow velocity increases by 66%, reflecting compensatory vasodilation, whereas skeletal muscle shows flow cessation in approximately 40% of capillaries with substantial slowing in the remainder [163]. This differential regional response reflects organ-specific priorities in oxygen allocation during systemic hypoxia and the distinct structural characteristics of microvascular networks in different tissues.

The regulation of hypoxic cerebral vasodilation involves a sophisticated carbon monoxide-dependent signalling cascade discovered by Mustafa et al. [164] Under normoxic conditions, constitutively generated carbon monoxide from heme oxygenase-2 inhibits cystathionine β-synthase. During hypoxia, heme oxygenase-2 activity decreases as it functions as an oxygen sensor, reducing carbon monoxide production and thereby releasing cystathionine β-synthase from inhibition. The resulting increase in hydrogen sulfide (H₂S) generation mediates vasodilation of precapillary arterioles, with this mechanism critical for maintaining CBF during oxygen deprivation. Genetic deletion studies confirm that both heme oxygenase-2 and cystathionine β-synthase are essential for normal hypoxic cerebrovascular responses [164], and mice lacking these enzymes show impaired ability to maintain ATP levels during hypoxia. Prostacyclin-induced vasodilation may also contribute to the normal hypoxic response, particularly when nitric oxide pathways are compromised [165]. 

Capillary recruitment represents another key adaptive mechanism. Under normoxic conditions in anesthetized rats, considerably less than 100% of cerebral capillaries actively perfuse tissue. During combined hypoxia and hypercapnia, maximum cerebral blood volume (12.1 ml/100 g) increases 3.6-fold compared to normoxic normocapnia, demonstrating substantial reserve capacity through capillary recruitment [166]. Mean capillary transit time reaches a minimum (1.1 s) with moderate hypoxia or hypercapnia, with combined stimuli producing much higher blood flow than either alone [166]. 

However, prolonged or severe hypoxia leads to microcirculatory pathology characterized by: (1) endothelial cell swelling secondary to increased membrane permeability, acidosis, and ATP depletion affecting ion pump function [165], (2) decreased release of vasodilatory mediators (prostacyclin, nitric oxide) coupled with increased release of vasoconstrictors (endothelin, thromboxane) [165], (3) reduced red blood cell deformability due to oxidative injury and ATP depletion [165], and (4) heterogeneous perfusion patterns with intermittently or non-perfused capillaries adjacent to well-perfused vessels [167]. This heterogeneity of perfusion creates localized zones of severe tissue hypoxia despite adequate global CBF, a phenomenon particularly relevant to understanding regional vulnerability in hypoxic-ischemic encephalopathy.

### Integration and clinical implications

The interplay between hypoxia, hypercapnia, acidosis, and microcirculatory dysfunction reveals a complex pathophysiological network in which these factors rarely operate in isolation. In clinical scenarios such as obstructive sleep apnoea, chronic obstructive pulmonary disease exacerbations, perinatal asphyxia, and cardiac arrest, all of these elements co-exist and interact. Understanding their combined effects is essential for both predicting outcomes and designing therapeutic interventions. In perinatal asphyxia, for instance, the condition encompasses hypoxia (decreased pO₂), hypercapnia (increased pCO₂), hypoxemia, metabolic acidosis (increased lactate), impaired blood gas exchange, and ischemia simultaneously [168]. 

Permissive hypercapnia, widely utilized as a ventilation strategy in intensive care settings, demonstrates how therapeutic manipulation of one component can influence the entire network. At PaCO₂ levels of 60–100 mmHg, permissive hypercapnia maintains CBF, preserves BBB integrity, reduces infarct size, and, when combined with mild to moderate hypoxia, provides synergistic neuroprotection [148, 169]. Zhou et al. demonstrated that hypercapnia produced more protective effects against hypoxia-ischemia-induced brain damage in rats with mild to moderate systemic hypoxia (PaO₂ >50 mmHg) than in rats with severe systemic hypoxia (PaO₂ <50 mmHg) [148]. The protective effect of CO₂ inhalation was associated with inhibition of hypoxia-induced disruption of cortical CBF and BBB permeability. However, this protective effect is dose-dependent and critically influenced by the severity of accompanying hypoxia; severe hypoxia abolishes the neuroprotective benefit, and excessive hypercapnia (PaCO₂ >100 mmHg) is itself deleterious [148]. 

The clinical translation of hypercapnic-hypoxic conditioning holds considerable promise. Clinical studies have already demonstrated efficacy in treating childhood cerebral palsy and diabetic polyneuropathy in children [149], and the approach requires fewer and shorter exposure sessions than traditional hypoxic conditioning alone; protective effects can be achieved after only three exposures compared to seven or more for hypoxic training [151]. The combination of brief respiratory conditioning protocols with pharmacological modulators of neuroprotective signalling pathways may offer an additive or even synergistic therapeutic strategy for neurological disorders characterized by chronic or recurrent hypoxia [149]. Recent work exploring the relationship between tolerance to combined hypoxia-hypercapnia and life expectancy suggests potential applications in longevity research [153]. 

The dose-response relationship is critical for therapeutic applications. Recent work on adaptive responses to hypoxia and hyperoxia emphasizes that the benefits of hypoxic conditioning depend on achieving the appropriate “dose” of hypoxic exposure [25]. Navarrete-Opazo and Mitchell have comprehensively reviewed how intermittent hypoxia can be therapeutic or pathological depending on exposure parameters, with therapeutic potential requiring careful dosing [39]. The integration of our understanding of hypoxia, hypercapnia, acidosis, and microcirculatory effects into a unified framework for dose optimization represents an important direction for future research.

Importantly, these findings must inform the interpretation of research on isolated mild hypoxia. While controlled studies of isolated hypoxia provide valuable mechanistic insights into specific adaptive pathways, such as HIF-1α signalling, angiogenesis, and neural stem cell regulation, their clinical applicability is limited by the reality that pathological hypoxia invariably involves additional metabolic and hemodynamic perturbations. The present review has focused on the specific effects of mild chronic hypoxia to delineate these mechanisms clearly, but clinicians and researchers must recognize that translating these findings to clinical practice requires consideration of the multifactorial nature of hypoxic conditions. Future research should increasingly focus on the integrated effects of these co-existing conditions to better reflect clinical reality and optimize therapeutic interventions, with particular attention to the emerging potential of combined hypoxic-hypercapnic conditioning as a more robust and clinically relevant neuroprotective strategy.

## Conclusions and future directions

The effects of mild chronic hypoxia are capable of inducing both adaptive responses that support brain function and pathological processes that cause neuronal damage. The outcome depends on multiple interacting factors, including oxygen concentration, exposure duration, and individual physiological characteristics.

A critical finding is the time-dependent nature of responses to mild hypoxia. Initial exposure triggers acute pathological changes, but over time, adaptive mechanisms emerge including HIF-1α stabilization, angiogenesis, and enhanced neuroplasticity. This temporal evolution from damage to adaptation underlies the ambiguous nature of mild chronic hypoxia and highlights the importance of considering exposure duration when evaluating its effects.

Methodological considerations are essential for interpreting research findings. In vitro cellular models establish fundamental mechanisms of hypoxic signalling but lack the systemic compensation, barrier filtering, and spatial heterogeneity present in living organisms. This discrepancy necessitates careful validation before extrapolating cellular findings to whole-organism physiology or pathophysiology.

At the cellular and vascular levels, mild hypoxia demonstrates capacity to enhance neural stem cell function, promote neurogenesis and gliogenesis, and drive cerebrovascular remodelling. However, these beneficial adaptations occur alongside transient BBB disruption and potential inflammatory activation. The balance between protective and harmful effects depends critically on hypoxia severity, with milder exposures generally favouring adaptation while more severe hypoxemia rapidly induces cellular damage.

Clinical contexts reveal that hypoxia rarely occurs in isolation. Accompanying conditions such as hypercapnia and acidosis substantially modify outcomes. The combination of mild hypoxia with moderate hypercapnia can provide synergistic neuroprotection through complementary molecular mechanisms. Understanding these interactions is essential for translating experimental findings to clinical applications.

Future research should prioritize establishing standardized definitions of mild chronic hypoxia across experimental systems to enable meaningful comparison of findings. Investigation of molecular mechanisms governing the transition from acute damage to chronic adaptation will illuminate therapeutic windows and intervention targets. The identification of biomarkers distinguishing beneficial from harmful hypoxic responses could enable personalized risk assessment and treatment monitoring. Additionally, rigorous evaluation of controlled hypoxic conditioning protocols in clinical trials is needed, with attention to dose-response relationships and patient selection.

The development of both pharmacological and non-pharmacological interventions represents important research directions. Understanding individual variability in hypoxic responses, including genetic and epigenetic factors, will be critical for clinical translation. Advanced neuroimaging techniques for early detection and monitoring of hypoxic changes will support these efforts.

In conclusion, mild chronic hypoxia represents a physiological paradox with potential for both harm and benefit. Advancing our understanding requires interdisciplinary collaboration to develop comprehensive research strategies that bridge experimental models and clinical applications. Recognizing the context-dependent nature of hypoxic responses will be essential for harnessing therapeutic potential while minimizing risks.

## Data Availability

No datasets were generated or analysed during the current study.

## Abbreviations

ARDS: Acute respiratory distress syndrome
ATF4: Activating transcription factor 4
AP-1: Activator protein 1
ADP: Adenosine diphosphate
ATP: Adenosine triphosphate
PAO₂: Alveolar oxygen pressure
AQP-4: Aquaporin-4
PaO₂: Arterial oxygen pressure
BBB: Blood–brain barrier
BSCB: Blood–spinal cord barrier
BCSFB: Brain-cerebrospinal fluid barrier
BDNF: Brain-derived neurotrophic factor
CO₂: Carbon dioxide
CBF: Cerebral blood flow
PtO₂: Cerebral tissue oxygen pressure
CSF: Cerebrospinal fluid
CNS: Central nervous system
CP: Choroid plexus
DAMPs: Damage-associated molecular patterns
DNA: Deoxyribonucleic acid
EL: Ependymal layer
EPO: Erythropoietin
GFAP: Fibrillary acidic protein
FGF21: Fibroblast growth factor 21
FSTL1: Follistatin-like 1
GLUT1: Glucose transporter 1
GLUT3: Glucose transporter 3
G-CSF: Granulocyte colony-stimulating factor
GM-CSF: Granulocyte–macrophage colony-stimulating factor
HR: Heart rate
hRSV: human respiratory syncytial virus
H₂S: Hydrogen sulfide
HIF-α: Hypoxia-inducible factor alpha
HIF-β: Hypoxia-inducible factor beta
HIF-1α: Hypoxia-inducible factor 1-alpha
HIF-2α: Hypoxia-inducible factor 2-alpha
HIFs: Hypoxia-inducible factors
iNOS: inducible nitric oxide synthase
PiO₂: Inspired oxygen pressure
IP-10: Interferon-γ-inducible protein 10
IL: Interleukin
LDHA: Lactate dehydrogenase A
LAT-1: L-type amino acid transporter 1
LIF: Leukemia inhibitory factor
mTOR: mechanistic target of rapamycin
mRNA: messenger ribonucleic acid
MMP9: Metalloproteinase 9
MMP: Matrix metalloproteinase
miRNA: microRNA
MAPK: Mitogen-activated protein kinase
MCP-3: Monocyte chemotactic protein-3
MSTN/GDF8: Myostatin / growth differentiation factor 8
NOX4: NADPH oxidase 4
NSC: Neural stem cell
NRP-1: Neuropilin-1
NLRP3: NLR family pyrin domain containing 3
NF-κB: Nuclear factor kappa B
O₂: Oxygen
pO₂: Oxygen partial pressure
SaO₂: Oxygen saturation
P-gp: P-glycoprotein
PaCO₂: Partial pressure of carbon dioxide
PAMPs: Pathogen-associated molecular patterns
PFK1: Phosphofructokinase-1
PI3K: Phosphoinositide 3-kinase
PHD: Prolyl hydroxylases
ROS: Reactive oxygen species
SPARC: Secreted protein acidic and rich in cysteine
SARS-CoV-2: Severe acute respiratory syndrome coronavirus 2
TLR2: Toll-like receptor 2
TNF-α: Tumor necrosis factor alpha
UPR: Unfolded protein response
VEGF: Vascular endothelial growth factor
XBP1: X-box binding protein 1
ZO-1: Zonula occludens-1
